# Text-Book of Hygiene

**Published:** 1891-08

**Authors:** 


TEXT-BOOK OF HYGIENE. A comprehensive treatise on the Principles and Practice of Preventive Medicine from an American standpoint. By George H. Rohé, M. D. Second edition, thoroughly revised and largely rewritten, with many illustrations and valuable tables. Philadelphia (1231 Filbert Street) and London. F. A. Davis, Publisher, 1890. Price, $2.50, net.

This book can be most highly commended. It treats of air, water, food, soil, sewage, house construction, hospitals, the hygiene of schools, industrial establishments, military camps, ships, and prisons. Also, bathing, clothing, disposal of dead, contagion, infection, quarantine, etc.

One of its most instructive chapters is on ground air and ground water, or a consideration of the air and water that permeate the ground, and the influences of these upon health. A careful study of this chapter will explain many mysterious causes of ill health, and enable the intelligent physician to suggest measures to correct them.

We observe with surprise (page 93) that the author does not disapprove of the feeding of milk-cows upon the refuse of breweries and distilleries. To his statements on this subject we take decided exception. To make beer, barley is moistened in water and kept warm until it begins to sprout. The diastase found at one end of the grain converts the starch which makes up the bulk of the grain into grape sugar. Hot water is then added to dissolve out this sugar. Yeast is added to this solution or "wort," and fermentation begins and converts it into beer. Now what is left of the grain after this procedure? Nothing but a hull, largely siliceous, thoroughly soaked with water, and ready for and even undergoing acetous fermentation which makes of it a "sour mash." Yet this rubbish is fed to cows, under the impression that it is food, and our author approves of it! Distillery "slop" is even worse, for the grain has been steeped in the liquid during the whole process of fermentation, and has then been run into the still, where it has been subjected to a boiling heat in order to separate the liquor which was formed by the fermenting process.

We recollect having once had the care of a baby two years old that was dying of marasmus. We first saw it in the summer time. After great effort we succeeded in rescuing the child, and it regained its health perfectly. In the winter it was taken violently ill with cholera infantum. We then discovered that it lived on milk that was taken from cows fed on brewery grains. There could be very little doubt that its sickness was caused by the kind of milk taken. A change in the milk at once ameliorated its symptoms. It is our opinion that every dairyman feeding such material to his cows and then selling the milk product should be severely punished.

There are many very interesting subjects dwelt upon in this book, but there is not time nor space in which to discuss them. Those interested will do well to procure the book and read for themselves. W. M. J.

